# Internal jugular vein variability predicts fluid responsiveness in cardiac surgical patients with mechanical ventilation

**DOI:** 10.1186/s13613-017-0347-5

**Published:** 2018-01-16

**Authors:** Guo-guang Ma, Guang-wei Hao, Xiao-mei Yang, Du-ming Zhu, Lan Liu, Hua Liu, Guo-wei Tu, Zhe Luo

**Affiliations:** Department of Critical Care Medicine, Zhongshan Hospital, Fudan University, No. 180 Fenglin Road, Shanghai, 200032 Xuhui District People’s Republic of China

**Keywords:** Internal jugular veins, Inferior vena cava, Stroke volume variation, Fluid responsiveness, Cardiac surgery

## Abstract

**Background:**

To evaluate the efficacy of using internal jugular vein variability (IJVV) as an index of fluid responsiveness in mechanically ventilated patients after cardiac surgery.

**Methods:**

Seventy patients were assessed after cardiac surgery. Hemodynamic data coupled with ultrasound evaluation of IJVV and inferior vena cava variability (IVCV) were collected and calculated at baseline, after a passive leg raising (PLR) test and after a 500-ml fluid challenge. Patients were divided into volume responders (increase in stroke volume ≥ 15%) and non-responders (increase in stroke volume < 15%). We compared the differences in measured variables between responders and non-responders and tested the ability of the indices to predict fluid responsiveness.

**Results:**

Thirty-five (50%) patients were fluid responders. Responders presented higher IJVV, IVCV and stroke volume variation (SVV) compared with non-responders at baseline (*P* < 0.05). The relationship between IJVV and SVV was moderately correlated (*r* = 0.51, *P* < 0.01). The areas under the receiver operating characteristic (ROC) curves for predicting fluid responsiveness were 0.88 (CI 0.78–0.94) for IJVV compared with 0.83 (CI 0.72–0.91), 0.97 (CI 0.89–0.99), 0.91 (CI 0.82–0.97) for IVCV, SVV, and the increase in stroke volume in response to a PLR test, respectively.

**Conclusions:**

Ultrasound-derived IJVV is an accurate, easily acquired noninvasive parameter of fluid responsiveness in mechanically ventilated postoperative cardiac surgery patients, with a performance similar to that of IVCV.

**Electronic supplementary material:**

The online version of this article (10.1186/s13613-017-0347-5) contains supplementary material, which is available to authorized users.

## Background

Fluid management is one of the most important treatments for stabilizing hemodynamics in patients after cardiac surgery. Hypovolemia may lead to inadequate organ perfusion, whereas fluid overload may lead to postoperative complications such as congestive heart failure or pulmonary edema [1–3]. In addition, patients who underwent cardiac surgery have a certain degree of myocardial stunning [4], and hence, caution should be taken regarding fluid management in patients with a limited cardiac reserve.

It is imperative to predict the patient’s fluid responsiveness before volume expansion [5]. Several parameters have been introduced in clinical practice to predict fluid responsiveness and to guide therapy [2]. Based on the influence of cycling intra-thoracic pressure on arterial pulse pressure or stroke volume, dynamic indicators such as arterial pulse pressure variation (PPV) or stroke volume variation (SVV) have been widely used as reliable predictors of fluid responsiveness [6–8]. However, these dynamic parameters have several limitations and can only be used under strict conditions.

Recently, noninvasive and point-of-care ultrasound seems to meet the criteria of an ideal bedside tool for fluid status assessment. Several studies have confirmed that respiratory variations of the superior and inferior vena cava diameters (collapsibility index [CI] and distensibility index [DI]) accurately reflect volume responsiveness in mechanically ventilated patients [9, 10]. Unfortunately, measurements of the inferior vena cava (IVC) and superior vena cava (SVC) may fail to predict fluid responsiveness following cardiac surgery due to methodological problems such as poor subcostal caval image quality caused by mediastinal air, surgical drains, dressings, abdominal distension or morbid obesity [11–13]; a more accurate measurement would require transoesophageal echocardiography (TEE). It is well known that pressure and volume changes within the intra-thoracic systemic venous compartment can transmit to the extrathoracic veins, for example, the intra-abdominal IVC or extrathoracic internal jugular vein (IJV) [14–16]. The IJV is, technically, much more easily accessible for sonographic visualization than the IVC, and measurement of the IJV does not require TEE. Internal jugular vein variability (IJVV) has been studied in several studies [17–19], but its reliability has not been well confirmed in patients after cardiac surgery. The aim of this study was to evaluate the reliability of IJVV, as visualized by ultrasound, to predict fluid responsiveness in mechanically ventilated patients after cardiac surgery.

## Methods

This study was approved by the Ethical Committee of Zhongshan Hospital affiliated to Fudan University (No. B2016077), and informed consent was obtained from all study participants. This trial has been registered at clinicaltrials.gov as NCT02852889.

## Patient selection

Patients who underwent cardiac surgery between August and December 2016 in the Cardiac Surgery Intensive Care Unit (CSICU) of the Zhongshan Hospital of Fudan University were screened for inclusion by research personnel. All patients routinely underwent a TEE during the operation and a postoperative (after admission to ICU and prior to study enrollment) comprehensive transthoracic echocardiography (TTE). The TEE was used to monitor the hemodynamics and confirm the postoperative effect of surgery. TTE was used to identify different causes of hypotension in postoperative period such as obstructive shock, hypovolemia and reduced ventricular systolic function. The patients were included when they presented with circulatory instability and required a rapid fluid challenge based on the clinical judgment of the attending physician. The physician’s decision was principally based on the presence of clinical signs of acute circulatory failure (low blood pressure or urine output, tachycardia, or mottling) and/or clinical signs of organ hypoperfusion (renal dysfunction or hyperlactatemia). The exclusion criteria included age < 18 years; evidence of cardiac arrhythmia (e.g., atrial fibrillation); evidence of jugular vein thrombosis; bilaterally inserted venous catheters (jugular or subclavian vein); echocardiographic examination that showed the existence of severe tricuspid or mitral regurgitation or right heart dysfunction (right ventricular fractional area change < 40% examined by TEE; tricuspid annular plane systolic excursion < 16 mm examined by TTE); a history of radiotherapy or surgery of the neck region or back (making it impossible to put the patient in a supine position with the head elevated to 30°); a contraindication to the passive leg raising (PLR) test; and the inability to obtain interpretable ultrasound images due to a difficult acoustic window.

All enrolled patients were sedated via propofol and morphine infusion, and with absence of inspiratory efforts according to the ventilator waveform and monitoring parameters. No muscular blocking agents were used in this study. All patients were ventilated in the intermittent positive pressure ventilation (IPPV) mode in the supine position with the head elevated to 30°. The ventilatory parameters were adjusted to the following criteria: tidal volume (Vt): 8 ml/kg predicted body weight (PBW), Pplat < 30 cmH_2_O, positive end-expiratory pressure (PEEP): 5 cmH_2_O, respiratory rate: 12–16 breaths per minute, PaCO_2_ ≤ 45 mmHg and oxygen saturation (SaO_2_) > 96%. The following baseline data were recorded for each patient: age (years), weight (kg), height (cm), diagnosis, type of cardiac surgery, acute physiology and chronic health evaluation (APACHE) II score, European system for cardiac operative risk evaluation (EuroSCORE), vasoactive drug infusion rates and preoperative echocardiographic parameters [left ventricular ejection fraction (LVEF), presence of left ventricular hypertrophy, right ventricular end-diastolic diameter, and tricuspid regurgitation grade].

## Measurements

We analyzed a series of measured hemodynamic variables from an indwelling radial arterial catheter and central venous catheter in each patient. These data included heart rate (HR) (beats/minute), mean arterial pressure (MAP) (mmHg), central venous pressure (CVP) (mmHg), stroke volume (SV) (ml), PLR-induced increase in stroke volume (PLR-ΔSV) (ml), and stroke volume variation (SVV) using the FloTrac/Vigileo (Edwards Lifesciences, Irvine, CA, USA) continuous hemodynamic monitoring system. The pressure transducers were consistently adjusted to the level of the patient’s right atrium.

Intensivists with a certification of ultrasound evaluation performed all of the ultrasound examinations. An associate critical care professor supervised the entire course of examinations. The intensivists performing the ultrasound examinations were blinded to the hemodynamic data. (These were collected by another investigator.) Sonographic measurements of the IJV and IVC diameters were taken using a Philips CX50 ultrasound device (Philips Healthcare, Hamburg, Germany) equipped with a linear transducer (L12-3 Broadband Linear Array Transducer) and a transthoracic phased array transducer (S5-1 Phased Array Transducer), respectively.

**Fig. 1:**
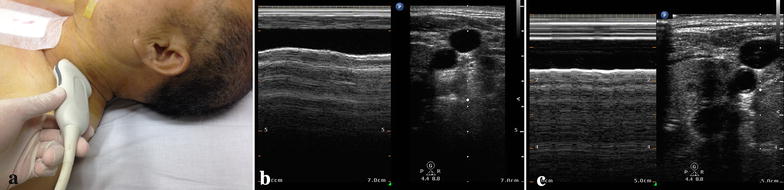
Ultrasound probe position for IJV detection at the cricoid cartilage level (**a**). The patient is in the supine position at 30°. M-mode assessment of the antero-posterior diameter of the IJV in a responsive patient (**b**, a high variability of IJV diameter is seen) and in a non-responsive patient (**c**, lack of variation of the IJV diameter is seen) while on mechanical ventilation

Patients admitted at the ICU after cardiac surgery had a conventional right IJV catheter. To avoid any risk of infection at the puncture site, sonographic measurements were taken on the left IJV. The IJV was visualized by placing the ultrasound transducer perpendicular to the skin in the transverse plane on the patient’s neck at the level of the cricoid cartilage in order to avoid interference from the probe-to-vein angle. The vein was identified by compression as well as by color Doppler imaging. To avoid any influence of external compression on the IJV diameter during the examination, sufficient ultrasound gel was used to prevent direct skin contact with the transducer [20], and thus, the least amount of pressure was applied (Fig. 1a).

An M-mode scan was recorded over a whole respiratory cycle (Fig. 1b, c), and then, the image was frozen. The maximum antero-posterior diameter of the IJV was measured at the end of inspiration [diamax (cm)], and the minimum diameter was measured at the end of expiration [diamin (cm)]. The IJV variability (IJVV) was calculated using the formula: IJVV (%) = (diamax − diamin)/[(diamax + diamin)/2] × 100. Using similar methods, the IVC was visualized longitudinally in the subxyphoid long-axis view, and its M-mode cursor was used to measure the IVC variability (IVCV) approximately 3 cm from the right atrium.

## Study design

Ultrasound examinations and the collection of hemodynamic data were performed at baseline (*T*0, in a supine position with the head elevated to 30° for baseline measurements), 1 min after a PLR test (*T*1, the bed was automatically moved to a position with the head elevated to 0° and the legs up to 45°) and after a 500-mL Gelofusine challenge (T2, the bed was returned to the initial position, and fluid was infused over 30 min). PLR was performed in order to compare the predictive value of different parameters in predicting fluid responsiveness. Vasoactive drug infusion rates and ventilation settings were kept constant during the study procedures. Patients were classified as “volume responders” if there was an increase in SV ≥ 15% after the fluid challenge, and the remaining patients were classified as “volume non-responders” [21, 22].

## Statistical analysis

The number of the enrolled patients was referred to similar studies evaluating the prediction ability of IJVV [17–19]. All continuous variables except the doses of norepinephrine and dobutamine were normally distributed (Kolmogorov–Smirnov test). The results are expressed as the mean ± SD (standard deviation) or median (25–75% inter-quartile range, IQR) as appropriate. After checking the homogeneity of variance for each parameter, the difference between values was compared using the independent sample *t* test, and the comparisons of hemodynamic variables between the different study times were assessed using paired Student *t* tests. Comparisons between responders and non-responders were assessed using two-sample Student’s *t* tests. *P* values < 0.05 were considered statistically significant. Correlations were assessed by Pearson coefficient. Receiver operating characteristic (ROC) curves were constructed to establish the sensitivity and specificity of dynamic and static indicators in predicting fluid responsiveness. The areas under the ROC curves (AUCs) were compared using DeLong and colleagues’ test. The optimal cutoff of each variable was estimated by maximizing the Youden index. A difference between two AUCs was considered statistically significant, when the *P* value of DeLong and colleagues’ test was < 0.05. Statistical analyses were performed with the MedCalc 8.1.0.0 (Mariakerke, Belgium) and SPSS software (19.0).

## Results

A total of seventy-five postoperative cardiac surgery patients were enrolled during a period of 5 months. Five patients were excluded because visualization of the IVC via ultrasound was technically difficult. Seventy patients (44 males and 26 females) were included in the final analysis. The reasons for hemodynamic instability were related to the hypovolemia (35 patients), cardiac dysfunction (27 patients) and vasoplegic shock (8 patients). The mean age of the patients was 61 ± 10 years, and the APACHE II scores were 9 ± 5. All patients were sedated and were in sinus rhythm. The patients’ mean LVEF (Simpson’s method) before surgery was 50%. Baseline patient characteristics and clinical data are shown in Table 1. Hemodynamic and ultrasound data in responders and non-responders at all study times [baseline (*T*0), during PLR (*T*1), and after fluid challenge (*T*2)] are reported in Table 2. Fluid challenge significantly increased SV by more than 15% in 35 (50%) patients (responders, from 39.87 ± 13.67 to 58.72 ± 22.16 ml, *P* < 0.05). The remaining 35 (50%) patients did not exhibit a significant change in SV (non-responders, from 49.86 ± 17.71 to 54.81 ± 16.53 ml). The results of PLR and fluid challenge in this study are shown in Additional file 1.

**Table 1:** Baseline characteristics of the patients (*n* = 70)

Characteristic	
Age (years)	61 ± 10
Male sex, *n* (%)	44 (62.86)
Body mass index (kg/m^2^)	22 ± 3
Left ventricular ejection fraction (%)	50
Cardiac surgery category, *n* (%)	
Valve	37 (52.86)
CABG	12 (17.14)
CABG + valve	7 (10.00)
Aortic surgery	9 (12.86)
Others	5 (7.14)
Postoperative day, *n* (%)	
*d*0	62 (88.57%)
*d*1	8 (11.43%)
APACHE II scores	9 ± 5
EuroSCORE	4 ± 2
Tidal volume (mL)	520 ± 28
PEEP (cm H_2_O)	5
PaO_2_/FiO_2_ (mmHg)	123 ± 57
Lactate (mmol/L)	3.23 ± 3.39
Patients receiving norepinephrine, *n* (%)	45 (64.29)
Patients receiving dobutamine, *n* (%)	9 (12.86)
Dose of norepinephrine (μg kg^−1^ min^−1^)	0.24 (0.15–0.35)
Dose of dobutamine (μg kg^−1^ min^−1)^	0.33 (0.28–0.43)

Values are expressed as mean ± SD, median (25–75% inter-quartile range) or number and frequency in %

*CABG* coronary artery bypass grafting, *APACHE II* acute physiology and chronic health evaluation, *EuroSCORE* European system for cardiac operative risk evaluation, *PEEP* positive end-expiratory pressure, *PaO*_*2*_ arterial partial pressure of oxygen, *FiO*_*2*_ inspiratory fraction of oxygen

**Table 2:** Hemodynamic parameters measured in responders and non-responders

	*T*0	*T*1	*T*2
HR (beats min^−1^)			
Responders	91 ± 20	89 ± 18	87 ± 14
Non-responders	88 ± 17	88 ± 17	87 ± 16
SBP (mmHg)			
Responders	87 ± 19	95 ± 29	119 ± 26^c^
Non-responders	111 ± 17^a^	116 ± 19^a^	112 ± 23
DBP (mmHg)			
Responders	46 ± 8	53 ± 7^b^	58 ± 8^c^
Non-responders	55 ± 11^a^	58 ± 9^a^	54 ± 8^a^
MAP (mmHg)			
Responders	58 ± 10	67 ± 9^b^	73 ± 11^c^
Non-responders	71 ± 10^a^	75 ± 10^a^	70 ± 9
CVP (mmHg)			
Responders	11 ± 4	11 ± 3	12 ± 3
Non-responders	12 ± 4	14 ± 4^a,b^	13 ± 4
CO (L/min)			
Responders	3.60 ± 1.54	4.68 ± 1.79^b^	5.11 ± 2.15^c^
Non-responders	4.17 ± 0.93	4.50 ± 1.17	4.69 ± 1.44
SV (ml)			
Responders	39.87 ± 13.67	52.99 ± 16.22^b^	58.72 ± 22.16^c^
Non-responders	49.86 ± 17.71^a^	53.52 ± 18.14	54.81 ± 16.53
SVV (%)			
Responders	14.94 ± 1.85	10.34 ± 5.26^b^	8.71 ± 4.59^c^
Non-responders	9.49 ± 2.67^a^	7.74 ± 4.83^a^	7.03 ± 2.67^c^
IJVV (%)			
Responders	23.04 ± 16.76	9.88 ± 13.76^b^	7.96 ± 8.72^c^
Non-responders	9.90 ± 5.63^a^	6.38 ± 2.37^b^	5.73 ± 2.02^c^
IVCV (%)			
Responders	15.97 ± 4.08	8.98 ± 4.52^b^	8.08 ± 7.70^c^
Non-responders	8.78 ± 5.42^a^	8.14 ± 4.94	6.41 ± 2.76^c^

Values are expressed as mean ± SD

*HR* heart rate, BP, *SBP* systolic blood pressure, *DBP* diastolic blood pressure, *MAP* mean arterial pressure, *CVP* central venous pressure, *CO* cardiac output, *SV* stroke volume, *SVV* stroke volume variation, *IJVV* internal jugular venous variability, *IVCV* inferior vena cava variability

*T0* baseline, *T1* after passive leg raising test, *T2* after fluid expansion

^a^*P* < 0.05 non-responders versus responders

^b^*P* < 0.05 T1 versus T0

^c^*P* < 0.05 T2 versus T0

Basal HR was not different between the responders and non-responders either at T1 or T2 (T1 89 ± 18 vs. 88 ± 17 beats min^−1^, T2 87 ± 14 vs. 87 ± 16 beats min^−1^), although HR tended to decrease after the PLR test or fluid challenge in responders. Responders displayed an increase in SBP, DBP and MAP from T0 to T2 (87 ± 19 vs. 119 ± 26 mmHg, *P* < 0.05; 46 ± 8 vs. 58 ± 8 mmHg, *P* < 0.05; and 58 ± 10 vs. 73 ± 11 mmHg, *P* < 0.05, respectively), and the same changes are also observed from T0 to T1 in DBP and MAP but not in SBP. No significant change in arterial pressure or HR was observed in non-responders. Non-responders generally displayed a higher CVP than responders after PLR (T1 14 ± 4 vs. 11 ± 3 mmHg, *P* < 0.05, Table 2). Although CVP tended to increase after the PLR test or fluid challenge in non-responders, we found a significant increase in CVP only after PLR (12 ± 4 vs. 14 ± 4 mmHg, *P* < 0.05); a difference was not observed after volume expansion (12 ± 4 vs. 13 ± 4 mmHg). In responders, a significant change in CVP was not observed after the PLR test nor the fluid challenge (11 ± 4 vs. 11 ± 3 mmHg; 11 ± 4 vs. 12 ± 3 mmHg).

**Fig. 2:**
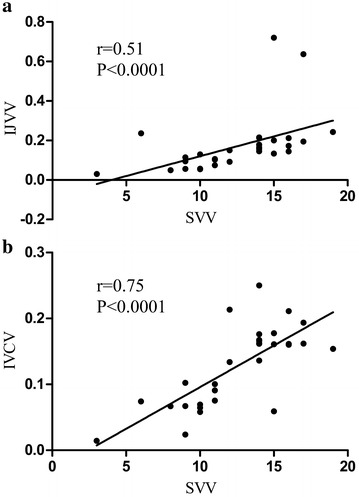
Pearson correlation analysis. (**a**, association between IJVV and SVV; **b**, association between IVCV and SVV)

In volume responders, IJVV, IVCV and SVV were significantly higher compared with non-responders at baseline. All of these values significantly decreased after the PLR test or fluid administration in responders. However, we found that both responders and non-responders exhibited a significant reduction in IJVV from baseline to the PLR test time or post-volume expansion, and similar findings were also presented for IVCV after fluid challenge (Table 2). We determined that the relationship between IJVV and SVV was moderately correlated (Fig. 2a, *r* = 0.51, *P* < 0.01). IVCV and SVV were significantly correlated (Fig. 2b, *r* = 0.75, *P* < 0.01).

**Fig. 3:**
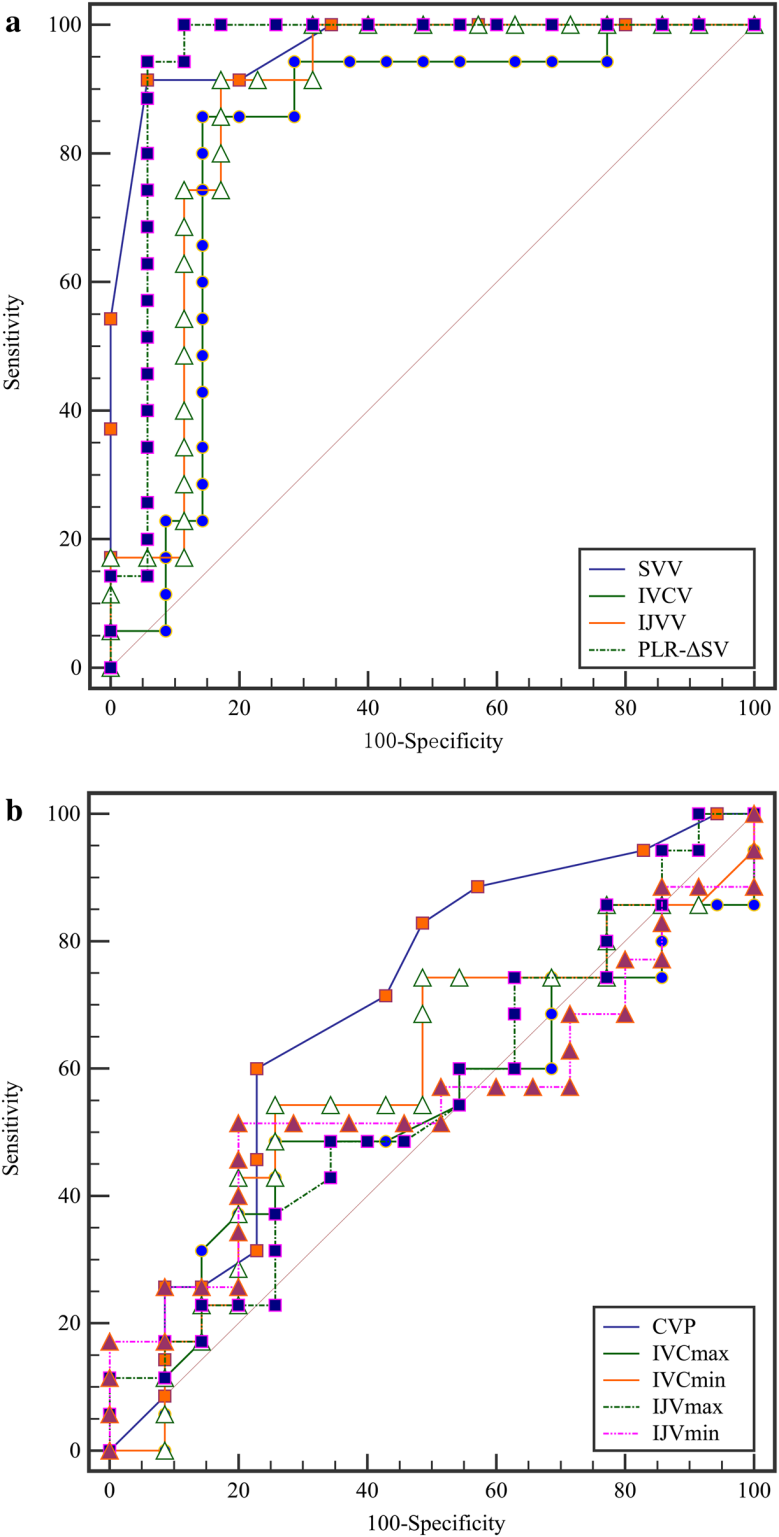
Comparison of the areas under the ROC curves for the indicators used for predicting fluid responsiveness (**a**, dynamic indicators; and **b**, static indicators)

The AUCs established for SVV and PLR-ΔSV were comparable (0.97 vs. 0.91, *P* = 0.61). The AUC of SVV was significantly greater than that of IVCV (0.97 vs. 0.83, *P* < 0.01) and IJVV (0.97 vs. 0.88, *P* = 0.03) (Fig. 3a). The AUCs for static indicators (CVP, IVC diameter and IJV diameter) were significantly lower than that of dynamic indicators (Fig. 3b). An SVV value > 12% was able to identify volume responders with a sensitivity of 91.43%, a specificity of 94.29% and an AUC of 0.97 (CI 0.89–0.99). The PLR-ΔSV > 12.84% for the prediction of fluid responsiveness was associated with a sensitivity of 100%, a specificity of 82.86% and an AUC of 0.91 (CI 0.82–0.97). IJVV > 12.99% predicted fluid responsiveness with a sensitivity of 91.43%, a specificity of 82.86% and an AUC of 0.88 (CI 0.78–0.94). IVCV showed an AUC of 0.83 (CI 0.72–0.91) with a cutoff value of 13.39% (sensitivity 85.71% and specificity 85.71%) (Table 3). A significant difference between IJVV and IVCV was not observed (0.88 vs. 0.83, *P* = 0.43).

**Table 3:** Diagnostic ability of the different indices of fluid responsiveness

	AUC (95% CI)	Optimal cutoff (%)	Sensitivity (%)	Specificity (%)	Youden index	Positive predictive value	Negative predictive value	Positive likelihood ratio	Negative likelihood ratio
Dynamic indicators									
SVV	0.97 (0.89–0.99)	12.00	91.43	94.29	0.86	0.94	0.92	16.00	0.09
PLR-ΔSV	0.91 (0.82–0.97)	12.84	100.00	82.86	0.83	0.85	1.00	5.83	0.00
IJVV	0.88 (0.78–0.94)	12.99	91.43	82.86	0.74	0.84	0.91	5.33	0.10
IVCV	0.83 (0.72–0.91)	13.39	85.71	85.71	0.71	0.86	0.86	6.00	0.17
Static indicators									
CVP	0.70 (0.57–0.80)	11.00	60.00	77.14	0.37	0.72	0.66	2.63	0.52
IVCmax	0.53 (0.40–0.65)	1.57	48.57	74.29	0.23	0.65	0.59	1.89	0.69
IVCmin	0.58 (0.46–0.70)	1.40	54.29	74.29	0.29	0.68	0.62	2.11	0.62
IJVmax	0.55 (0.43–0.67)	0.86	48.57	65.71	0.14	0.59	0.56	1.42	0.78
IJVmin	0.55 (0.43–0.67)	0.64	51.43	80.00	0.31	0.72	0.62	2.57	0.61

*AUC* area under the receiver operating characteristic curve, *CI* confidence interval, *SVV* respiratory variation of stroke volume, *PLR*-∆*SV* the increase in stroke volume in response to a passive leg raising test, *IJVV* internal jugular venous variability, *IVCV* inferior vena cava variability, *CVP* central venous pressure, *IVCmax* the maximum inferior vena cava diameter, *IVCmin* the minimum inferior vena cava diameter, *IJVmax* the maximum internal jugular venous diameter, *IJVmin* the minimum internal jugular venous diameter

The intra-observer variability and inter-observer variability of IJVV measurement were further investigated in 30 patients. The results showed good concordance between estimation of IJVV by the two investigators, with a mean bias of − 0.01 and limits of agreement between − 0.1 and 0.08. The reliability of the measurements was also analyzed with intraclass correlation coefficients (ICCs) assessing intra-observer and inter-observer correlation (Additional file 2).

## Discussion

The objective of this study was to evaluate whether ultrasound assessment of IJV respiratory diameter changes can serve as a simple indicator of fluid responsiveness in mechanically ventilated patients after cardiac surgery. Our data showed that IJVV was comparable to IVCV in predicting fluid responsiveness. There was a positive correlation between SVV and ventilator-induced IJVV. It was also found that the predictive value of PLR-ΔSV and SVV was superior to that of IVCV and IJVV.

Correcting hypovolemia is of paramount importance during the postoperative critical care of cardiac surgical patients. However, its correction should be carefully guided to avoid unnecessary volume expansion [23]. Therefore, many investigators have explored reliable techniques with the goal of predicting fluid responsiveness in critically ill patients. Static parameters, such as CVP, are poor predictors of fluid responsiveness as previously reported and as shown in our study [23–25]. Based on the hemodynamic consequences of the heart–lung interactions, the use of dynamic indices of preload that result from respiratory variations is well-accepted bedside parameters of fluid responsiveness [7]. It was worth mentioning that tidal volume should be large enough to promote adequate preload variations. Fluid responsiveness cannot be reliably predicted if the tidal volume is < 8 ml/kg PBW [26]. Therefore, a Vt 8 mL/kg PBW was set in the present study. As higher PEEP may have adverse effects such as overinflation and hemodynamic deterioration, a PEEP of 5 cm H_2_O was set initially after cardiac surgery according to our routine practice.

Mechanical ventilation-induced cyclic variations in vena cava diameter have been shown to be accurate predictors of fluid responsiveness. In our study, we have shown that the IVCV was a good predictor of fluid responsiveness for mechanically ventilated patients following cardiac surgery. IVCV threshold values of 13.39% have been reported in the literature to be able to discriminate between responders and non-responders with a sensitivity of 85.71% and a specificity of 85.71%. Based on the associations of intra-thoracic venous pressure and volume with extrathoracic venous pressure, we hypothesized that fluid responsiveness may also be reflected by changes in IJV pressure as assessed by IJVV. Measuring IJV diameter change is easily achieved with ultrasound with minimal training, as this approach is frequently used for ultrasound-guided central vein catheterization. We demonstrated the reliability of IJVV with a value of 12.99% in detecting fluid responsiveness, having a sensitivity of 91.43% and a specificity of 82.86% in mechanically ventilated cardiac surgical patients.

Several studies have investigated the ability of respiratory variations in IJV diameter to evaluate hypovolemia or a hemodynamic response to a fluid challenge. Guarracino et al. have reported that IJV distensibility [(diamax − diamin)/diamin × 100] accurately predicts volume responsiveness in mechanically ventilated septic patients [19]. A cutoff value of 18% IJV distensibility resulted in 80% sensitivity and 85% specificity for predicting a fluid response, which was defined as an increase in cardiac index ≥ 15%. However, this study did not include patients with cardiac disease who have different hemodynamic characteristics. Moreover, the authors did not compare the predictive values of IVCV and IJVV. Thudium et al. showed that ultrasound evaluation of IJV extensibility can change in response to preload-altering orthostatic maneuvers and pulse pressure variation alterations [17]. However, this study was conducted at the cardiac surgery intensive care unit, and all of the patients were included after elective cardiac surgery; the reporters did not perform the standard fluid challenge, and the subgroup analysis showed that different surgery categories had different results. Broilo et al. verified the hypothesis that respiratory variations of the IVC and IJV were correlated [18]. These two indicators showed a significant agreement in evaluating fluid responsiveness. However, they did not identify changes in cardiac output following a fluid challenge, and they did not evaluate changes in the vein diameters before and after a fluid challenge. There were other studies demonstrating its utility, using measurements of the IJV to detect early hemorrhage in healthy volunteers that were donating blood [27, 28]. To our knowledge, this was the first study to evaluate the value of IJVV in predicting fluid responsiveness based on a standard fluid challenge in mechanically ventilated cardiac surgical patients.

Our study has several limitations. First, all subjects were on mechanical ventilation and absence of spontaneous breathing under sedation. Whether the conclusions can be extrapolated to patients with spontaneous breathing remains uncertain. Second, an uncalibrated system for hemodynamic monitoring was used in this study. Although the validation of FloTrac/vigileo system in measuring cardiac output has been assessed by numerous studies, the reliability of uncalibrated devices is still under debate [29–31]. Compared with pulmonary artery catheter (PAC) or transpulmonary thermodilution devices, FloTrac/vigileo system can be directly connected to the arterial catheter and has the advantage of auto-calibration. It theoretically meets the needs for rapidly assessing hemodynamic changes. Moreover, the dynamic indicator of SVV that could continuously displayed by the FloTrac/Vigileo system has also been shown to be able to predict fluid responsiveness in cardiac surgical patients [32–34]. Third, we did not enroll patients with right heart failure, as severe right heart failure or high CVP could influence IJV pressure and diameter and may decrease the relative variability even in the presence of preload responsiveness. Fourth, technical errors were possible, because even a slight pressure could have caused a great change in the cross-sectional image and diameter of the IJV during the acquisition of the measurements. We have made further efforts on the reproducibility and agreement of IJVV in 30 patients. The results showed good concordance between estimation of IJVV by the two investigators. Fifth, the initial semirecumbent position of the patient was 30° head of the bed (HOB) elevated instead of 45° (standard baseline position of PLR), because this was the recommended position for supine ventilated patient in the ICU. It was believed that this was more consistent with clinical scenario. Furthermore, taking sonographic measurements of the IVC diameters seems more easily in the position with HOB 30° than HOB 45°. The predict value of IJVV in other positions (such as the horizontal position) remains to be assessed.

## Conclusions

Ultrasound evaluation of IJVV is a simple, easy and readily accessible bedside measurement that predicts volume responsiveness in mechanically ventilated cardiac surgical patients. The respiratory variations of the IJV and IVC showed comparable value in the prediction of fluid responsiveness.

## Additional files

**Additional file 1.** The results of PLR and fluid challenge in this study.

**Additional file 2.** The reproducibility and agreement of IJVV in 30 patients.

## Abbreviations

HR: Heart rate
MAP: Mean arterial pressure
CVP: Central venous pressure
SV: Stroke volume
SVV: Stroke volume variation
IJV: Internal jugular venous
IJVV: Internal jugular venous variability
IVC: Inferior vena cava
IVCV: Inferior vena cava variability
PLR: Passive leg raising
PLR-ΔSV: The increase in stroke volume in response to a passive leg raising test
CSICU: Cardiac surgery intensive care unit
PEEP: Positive end-expiratory pressure
PBW: Predicted body weight
IPPV: Intermittent positive pressure ventilation
LVEF: Left ventricular ejection fraction
ROC: Receiver operating characteristic
