# Supplementary material for: Internal jugular vein variability predicts fluid responsiveness in cardiac surgical patients with mechanical ventilation
Source: Ann Intensive Care. 2018 Jan 16;8:6. doi: 10.1186/s13613-017-0347-5 (PMC5770347; doi:10.1186/s13613-017-0347-5)
Supplement: Supplementary file 1 — Additional file 1. The results of PLR and fluid challenge in this study. [file 13613_2017_347_MOESM1_ESM.docx]

**Additional file 1**

The results of PLR and fluid challenge in this study were shown as following

|  | Fluid challenge (+) | Fluid challenge (-) |
| --- | --- | --- |
| PLR (+) | 33 | 4 |
| PLR (-) | 2 | 31 |
