# Supplementary material for: Internal jugular vein variability predicts fluid responsiveness in cardiac surgical patients with mechanical ventilation
Source: Ann Intensive Care. 2018 Jan 16;8:6. doi: 10.1186/s13613-017-0347-5 (PMC5770347; doi:10.1186/s13613-017-0347-5)
Supplement: Supplementary file 2 — Additional file 2. The reproducibility and agreement of IJVV in 30 patients. [file 13613_2017_347_MOESM2_ESM.docx]

**Additional file 2**

The reproducibility and agreement of IJVV in 30 patients.

**Results**

**Table 1** Measurements of IJVV by observer A

| observer A | | | | | | |
| --- | --- | --- | --- | --- | --- | --- |
| Patients | 1st | | | 2nd | | |
|  | diamax | diamin | IJVV | diamax | diamin | IJVV |
| 1 | 1.11 | 1.02 | 0.0845 | 1.115 | 1.016 | 0.0929 |
| 2 | 1.02 | 0.65 | 0.4431 | 1.025 | 0.638 | 0.4654 |
| 3 | 0.967 | 0.956 | 0.0114 | 0.968 | 0.954 | 0.0146 |
| 4 | 0.678 | 0.533 | 0.2395 | 0.68 | 0.538 | 0.2332 |
| 5 | 1.03 | 0.889 | 0.1470 | 1.033 | 0.89 | 0.1487 |
| 6 | 1.28 | 1.09 | 0.1603 | 1.283 | 1.094 | 0.1590 |
| 7 | 1.01 | 0.922 | 0.0911 | 1.013 | 0.922 | 0.0941 |
| 8 | 1.03 | 0.811 | 0.2379 | 1.028 | 0.812 | 0.2348 |
| 9 | 1.04 | 1.01 | 0.0293 | 1.038 | 1.011 | 0.0264 |
| 10 | 0.889 | 0.833 | 0.0650 | 0.889 | 0.831 | 0.0674 |
| 11 | 1.23 | 1.17 | 0.0500 | 1.232 | 1.175 | 0.0474 |
| 12 | 0.822 | 0.644 | 0.2428 | 0.826 | 0.643 | 0.2491 |
| 13 | 0.733 | 0.667 | 0.0943 | 0.738 | 0.669 | 0.0981 |
| 14 | 0.933 | 0.832 | 0.1144 | 0.936 | 0.83 | 0.1200 |
| 15 | 0.822 | 0.644 | 0.2428 | 0.823 | 0.64 | 0.2502 |
| 16 | 0.825 | 0.645 | 0.2449 | 0.826 | 0.642 | 0.2507 |
| 17 | 0.765 | 0.69 | 0.1031 | 0.768 | 0.7 | 0.0926 |
| 18 | 0.822 | 0.644 | 0.2428 | 0.83 | 0.64 | 0.2585 |
| 19 | 0.757 | 0.611 | 0.2135 | 0.761 | 0.612 | 0.2170 |
| 20 | 1.77 | 1.67 | 0.0581 | 1.773 | 1.676 | 0.0562 |
| 21 | 1.1 | 1.02 | 0.0755 | 1.107 | 1.024 | 0.0779 |
| 22 | 1.7 | 1.59 | 0.0669 | 1.71 | 1.61 | 0.0602 |
| 23 | 1.4 | 1.2 | 0.1538 | 1.43 | 1.2 | 0.1749 |
| 24 | 2.46 | 2.3 | 0.0672 | 2.45 | 2.33 | 0.0502 |
| 25 | 1.95 | 1.76 | 0.1024 | 1.98 | 1.77 | 0.1120 |
| 26 | 0.6 | 0.567 | 0.0566 | 0.607 | 0.56 | 0.0805 |
| 27 | 0.822 | 0.644 | 0.2428 | 0.828 | 0.646 | 0.2469 |
| 28 | 0.86 | 0.74 | 0.1500 | 0.851 | 0.732 | 0.1503 |
| 29 | 0.822 | 0.644 | 0.2428 | 0.838 | 0.641 | 0.2664 |
| 30 | 0.733 | 0.578 | 0.2365 | 0.728 | 0.57 | 0.2435 |

**Table 2** Measurements of IJVV by observer B

| Patients | observer B | | |
| --- | --- | --- | --- |
|  | diamax | diamin | IJVV |
| 1 | 1.09 | 1.02 | 0.0664 |
| 2 | 1.04 | 0.67 | 0.4327 |
| 3 | 0.911 | 0.900 | 0.0121 |
| 4 | 0.644 | 0.500 | 0.2517 |
| 5 | 0.867 | 0.611 | 0.3464 |
| 6 | 1.12 | 0.911 | 0.2058 |
| 7 | 0.988 | 0.922 | 0.0691 |
| 8 | 1.16 | 0.878 | 0.2767 |
| 9 | 1.12 | 1.09 | 0.0271 |
| 10 | 1.02 | 0.933 | 0.0891 |
| 11 | 1.3 | 1.2 | 0.0800 |
| 12 | 0.88 | 0.68 | 0.2564 |
| 13 | 0.745 | 0.67 | 0.1060 |
| 14 | 1 | 0.85 | 0.1622 |
| 15 | 0.825 | 0.645 | 0.2449 |
| 16 | 0.828 | 0.644 | 0.2500 |
| 17 | 0.79 | 0.71 | 0.1067 |
| 18 | 0.85 | 0.68 | 0.2222 |
| 19 | 0.8 | 0.68 | 0.1622 |
| 20 | 1.8 | 1.7 | 0.0571 |
| 21 | 1.15 | 1.09 | 0.0536 |
| 22 | 1.85 | 1.62 | 0.1326 |
| 23 | 1.45 | 1.23 | 0.1642 |
| 24 | 2.5 | 2.33 | 0.0704 |
| 25 | 1.99 | 1.8 | 0.1003 |
| 26 | 0.63 | 0.57 | 0.1000 |
| 27 | 0.85 | 0.65 | 0.2667 |
| 28 | 0.9 | 0.8 | 0.1176 |
| 29 | 0.85 | 0.66 | 0.2517 |
| 30 | 0.741 | 0.623 | 0.1730 |

**Statistical analysis**

Bland-Altman analysis showed good concordance between estimation of IJVV by the two investigators, with a mean bias of -0.01 and limits of agreement between -0.1 and 0.08. (Fig. 1)


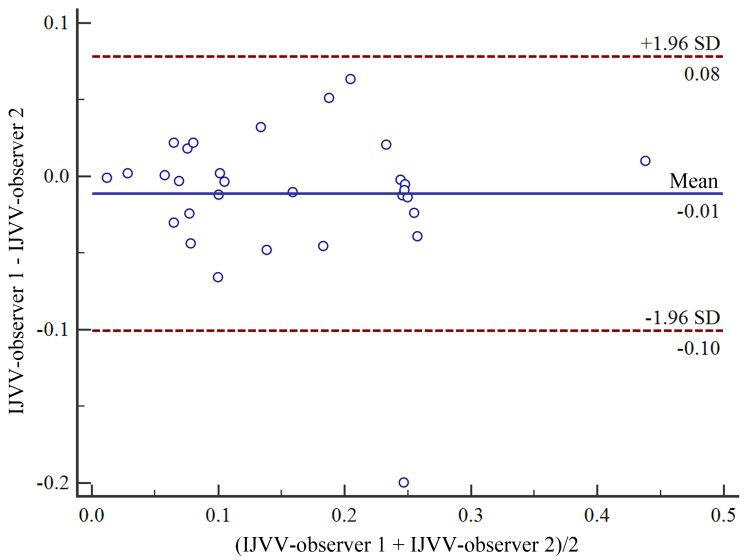


**Fig.1** Reproducibility and agreement of IJVV

The reliability of the measurements was also analyzed with intraclass correlation coefficients (ICCs) assessing intra-observer and inter-observer correlation. (Table 3 and 4)

**Table 3** Intraobserver reliability

|  | Cronbach α | ICC | 95% CI | Significance  (P value) |
| --- | --- | --- | --- | --- |
| diamin | 1.000 | 1.000 | 1.000-1.000 | <0.001 |
| diamax | 1.000 | 1.000 | 1.000-1.000 | <0.001 |
| IJVV | 0.998 | 0.995 | 0.990-0.998 | <0.001 |

**Table 4** Interobserver reliability

|  | Cronbach α | ICC | 95% CI | Significance  (P value) |
| --- | --- | --- | --- | --- |
| diamin | 0.992 | 0.985 | 0.968-0.993 | <0.001 |
| diamax | 0.994 | 0.987 | 0.973-0.994 | <0.001 |
| IJVV | 0.944 | 0.893 | 0.788-0.948 | <0.001 |
